# Supplementary material for: An Examination of Perceptions among Black Women on Their Awareness of and Access to Pre-Exposure Prophylaxis (PrEP)
Source: Int J Environ Res Public Health. 2024 Aug 16;21(8):1084. doi: 10.3390/ijerph21081084 (PMC11354061; doi:10.3390/ijerph21081084)
Supplement: Supplementary file 1 [file ijerph-21-01084-s001.zip › ijerph-3103540-supplementary.pdf]

PrEP-eligible Cis Gender Black Women

Focus Group Tool

Introduction Script for Focus Groups.

Good day Beautiful Black Women. I am Shadawn McCants and I will be the facilitator for your focus group discussion today. You have consented to participate in this focus group alongside other eligible Black women to discuss your thoughts on pre-exposure prophylaxis (PrEP).

The data indeed show that the disproportionate impacts of HIV for Black women are consistent, historic and undeniable. Black women comprise 55% of new HIV diagnoses among cisgender women and 46% of new diagnoses among transgender women, despite making up less than 15% of the female population. Black women continue to be diagnosed late—21% with a concurrent HIV and AIDS diagnosis—and are often shocked when they receive their diagnosis. This data is particularly striking in the face of breakthroughs in prevention options over the last decade, including ones that can be controlled by women.

The gap between PrEP-eligibility and PrEP access and uptake among cisgender Black women is glaring. Principal Investigator, Dr. Mandy Hill, led our team to develop a pilot grant, funded by the Ujima Program Scholars program funded by the NIH, to assess perspectives from cisgender Black women. We want to know how we can help bridge the gap to PrEP among PrEP-eligible cis Black women. We are hopeful that the findings of this focus group will help us to design intervention strategies that can help eligible cis Black women consider PrEP as an HIV prevention option that will contribute to their overall sexual health and reproductive health. We will now begin with the questions for the interview.

FCG Tool

1. What would prompt you to get an HIV test?
2. Do you believe that you are at risk for HIV? Why or why not?
3. What is PrEP?
4. What have you heard about PrEP before today?
5. Where have you heard about PrEP?
6. Is PrEP an option for you?
    a. Why or why not?
7. What do you know about PrEP?
8. How would you know if PrEP is for you?
9. Who is PrEP for?
10. What are some reasons why women would choose to use PrEP to prevent HIV?
11. What are some reasons why women would choose NOT to use PrEP to prevent HIV?
12. What are some reasons women would suggest that their partner should use PrEP?
13. Do you know of anyone in your social network who has previously used PrEP or is currently on PrEP?
14. When you see advertisements and marketing about PrEP on different media outlets, how do you feel PrEP is portrayed to you?
15. Do you feel represented in the way PrEP is portrayed in the media and on social media?
16. What kind of content in marketing and advertisements would prompt you to consider PrEP for yourself?
17. What are some of your primary concerns regarding PrEP?

18. What might be some reasons that cisgender Black women may or may not take the PrEP pill each day?
19. What might be some reasons that cisgender Black women may or may not take the PrEP as an injection (shot), taken every two months?
20. What are some of the concerns you have regarding the possible side effects of taking PrEP?
21. Do you believe the side effects of PrEP may outweigh its benefits? Why or why not?
22. What do you think would/could happen if others knew about your interest/use of PrEP?
23. How could a healthcare provider help you to feel more comfortable accessing PrEP?
24. What is your preference when choosing a healthcare provider to speak to about sexual health?
    a. Does discipline matter to you? Physician, Nurse, Social Worker?
    b. Does the way they communicate with you matter to you?
    c. What do you think about racial/ethnic, age, and gender identity? Do you feel more comfortable with someone that is a woman or a Black woman? Someone your age? Why or why not?
25. What has your experience been like when discussing your sexual health with a healthcare provider?
26. What would trust in the healthcare system look like for you?
27. What would provider trust look like for you?
28. Do you have access to reliable transportation?
    a. Does this access facilitate PrEP uptake for you? Why or why not?
29. What do you think can be done to help women take PrEP?
    a. What do you think can be done to help women to take PrEP daily or receive a bi-monthly injection, as prescribed?
30. Do you feel that adding PrEP as an injection into your routine every few months would be realistic with your current schedule?
31. Do you think PrEP can allow more sexual autonomy and fluidity? Why or why not?
32. How would you link PrEP uptake to showing yourself love and respect?
33. How could PrEP use improve the intimacy in your relationships?
34. What does 'feeling empowered' in your sex life look like to you?
